# Restoration of anatomical continuity after spinal cord transection depends on Wnt/β-catenin signaling in larval zebrafish

**DOI:** 10.1016/j.dib.2017.10.068

**Published:** 2017-11-04

**Authors:** Daniel Wehner, Thomas Becker, Catherina G. Becker

**Affiliations:** Centre for Neuroregeneration, University of Edinburgh, The Chancellor's Building, 49 Little France Crescent, Edinburgh EH16 4SB, UK

**Keywords:** Wnt, Beta-catenin, Regeneration, Spinal cord, Zebrafish

## Abstract

This data article contains descriptive and experimental data on spinal cord regeneration in larval zebrafish and its dependence on Wnt/β-catenin signaling. Analyzing spread of intraspinally injected fluorescent dextran showed that anatomical continuity is rapidly restored after complete spinal cord transection. Pharmacological interference with Wnt/β-catenin signaling (IWR-1) impaired restoration of spinal continuity. For further details and experimental findings please refer to the research article by Wehner et al. Wnt signaling controls pro-regenerative Collagen XII in functional spinal cord regeneration in zebrafish (Wehner et al., 2017) [1].

**Specifications Table**

**Table:** 

Subject area	Neurosciences
More specific subject area	Regenerative Biology
Type of data	Graph, Figure
How data was acquired	Confocal microscope (Zeiss LSM 780, Zeiss LSM 880) Fluorescent Stereo Microscope (Leica MZ16F, camera: DFC300FX)
Data format	Analyzed data, Processed data
Experimental factors	Restoration of anatomical continuity following complete spinal cord transection was studied in zebrafish larvae. The effect of Wnt/β-catenin pathway inhibition on spinal cord regeneration was analyzed.
Experimental features	Spread of intraspinally injected fluorescent dextran was analyzed to assess restoration of anatomical continuity of the spinal cord after complete transection in zebrafish larvae with and without inhibition of the Wnt/β-catenin pathway. Dextran distribution was assessed in live larvae or in transverse sections of fixed animals using fluorescence microscopy.
Data source location	Centre for Neuroregeneration, University of Edinburgh, United Kingdom
Data accessibility	The data are available with this article.

**Value of the data**

• Intraspinal injection of fluorescently-labelled dextran allows for labelling of whole spinal cord tissue in living zebrafish larvae.
• This method can be used to assess restoration of anatomical continuity after complete spinal cord transection.
• The data show that zebrafish larvae rapidly restore anatomical continuity after complete spinal cord transection and that this process depends on Wnt/β-catenin pathway activity.

## 1 Data

**Fig. 1:**
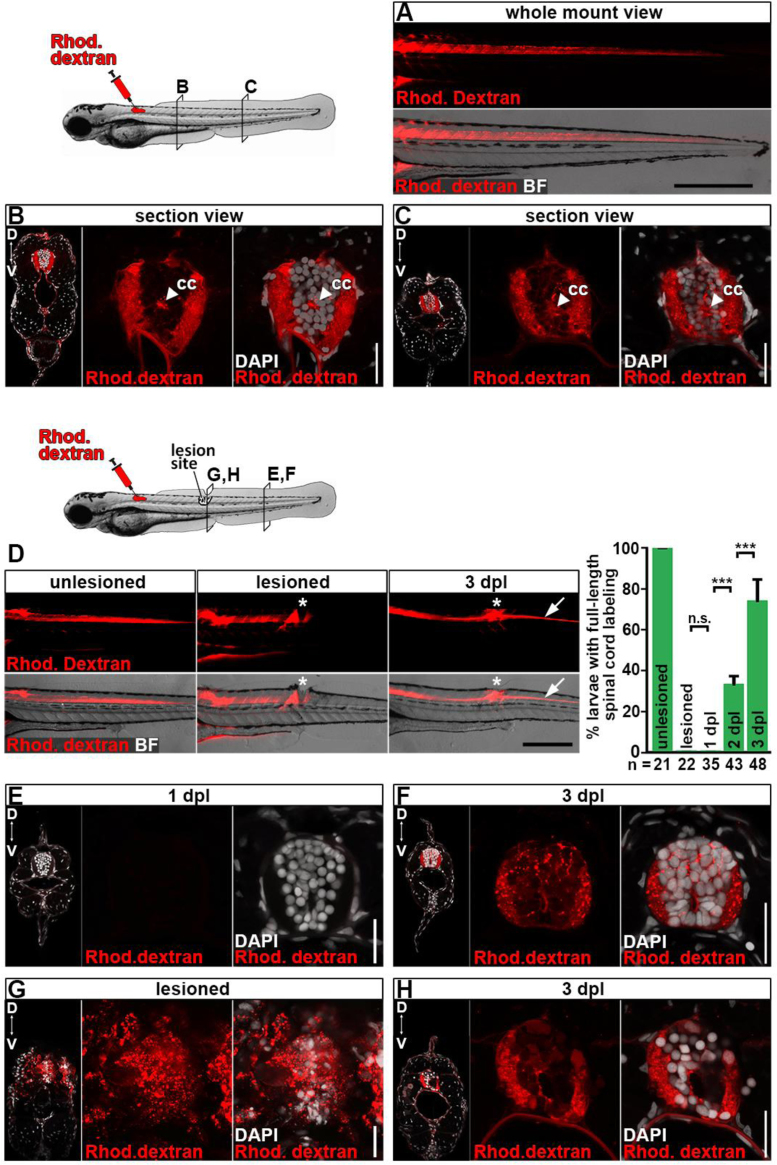
The spinal cords structurally reconnect after transection in zebrafish larvae. A-C) Intraspinal injection of fluorescent dextran into the rostral end of the spinal cord distributes to the whole spinal cord tissue, including neuropil and central canal, along the entire rostro-caudal axis. Shown is a lateral view of a whole mount larva (A) and transverse sections through the larva at different positions along the rostro-caudal axis (B-C). Approximate positions of the sections are indicated in the cartoon. **D-H)** Distribution of intraspinally injected dextran at different time points post-lesion. Fluorescent dextran injection fails to label spinal cord tissue caudal to the lesion site in freshly lesioned animals and at 1 dpl (D). At 2 dpl 33% of dextran-injected larvae show spinal cord labeling caudal to the lesion site (arrow), which further increases to 74% at 3 dpl, indicating restoration of spinal tissue continuity (D). Asterisk indicates lesion site. Fischer's exact test: ***P<0.001, n.s. indicates not significant. E) Transverse section of a 1 dpl larva shows absence of dextran labelling of spinal cord tissue caudal to the lesion site. F) Transverse section of a 3 dpl larva shows dextran labeling of spinal cord tissue caudal to the lesion site. G) Transverse section through the lesion site immediately after lesion shows dextran labelling of tissue surrounding the spinal cord. H) Transverse section through the lesion site at 3 dpl shows that dextran labelling is largely confined to regenerated spinal cord tissue. Approximate positions of the sections are indicated in the cartoon. **A-H)** Views are lateral (A, D; dorsal is up, rostral is left) or transversal (sections in B-C, E-H; dorsal is up). Abbreviations: BF, brightfield; Rhod. Dextran, Rhodamine dextran; cc, central canal. Scale bars: 250 µm (A), 200 µm (D), 25 µm (B-C, F), 20 µm (E, G-H). Error bars indicate s.e.m.

**Fig. 2:**
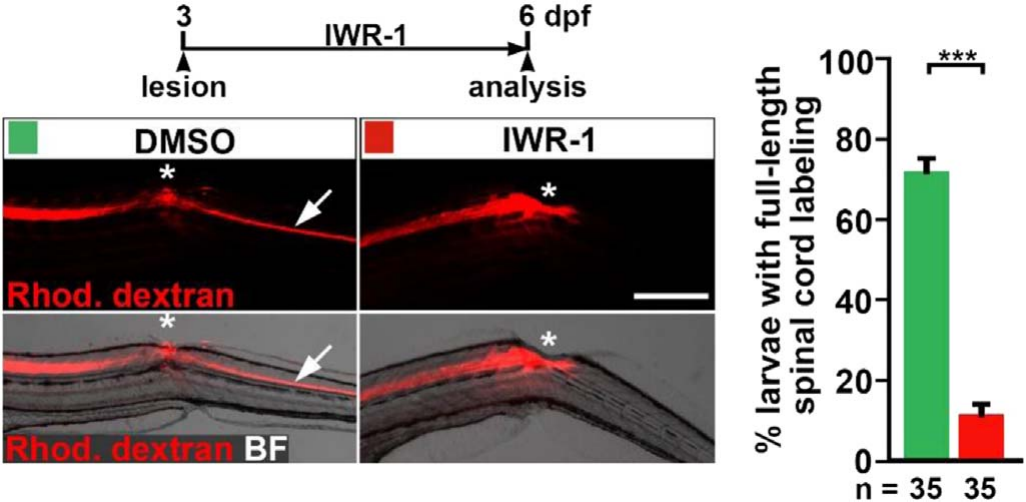
Inhibition of Wnt/β-catenin signaling inhibits dye spreading towards the caudal transected spinal cord. Pharmacological inhibition of the Wnt/β-catenin pathway (IWR-1 treatment) reduces the proportion of animals with dextran transport beyond the spinal lesion site, indicating lack of restored spinal continuity (Fischer's exact test: ***P<0.001). In DMSO-treated control larvae, intraspinally injected fluorescent dextran is transported across the lesion site (asterisk) into the caudal spinal cord (arrow). Dextran transport is interrupted at the level of the lesion site (asterisk) in IWR-1-treated larvae. Shown is a lateral view, dorsal is up, rostral is left). Abbreviations: BF, brightfield; Rhod. Dextran, Rhodamine dextran. Scale bar: 200 µm. Error bars indicate s.e.m.

Recently, we developed a larval zebrafish spinal cord injury model, which involves mechanical transection of the spinal cord at 3 days post-fertilization (dpf) [1], [2]. To assess whether ends of transected spinal cords structurally re-connect after a lesion, that is restoration of anatomical continuity, we assayed spreading along the spinal cord of fluorescently-labelled dextran that had been directly injected into the rostral end of the spinal cord. In unlesioned larvae, dextran was readily distributed through central canal and neuropil across the entire rostro-caudal axis of the spinal cord within less than a minute (Fig. 1A-C). Analysing transverse sections through the larva at different rostro-caudal positions confirmed that dextran labelling was confined to the spinal cord (Fig. 1B-C). In freshly lesioned animals and at 1 day post-lesion (dpl), injected dextran failed to distribute to spinal cord tissue caudal to the lesion site (Fig. 1D, E). At 2 dpl, we observed spinal cord labelling caudal to the lesion site in 33% of injected larvae. This proportion further increased to 74% at 3 dpl (Fig. 1D). Transverse sections of larvae at 3 dpl confirmed dextran spreading across the lesion site into the caudal spinal cord, but not into surrounding tissue (Fig. 1F). In the lesion site, dextran labelling was largely confined to regenerated spinal cord tissue at 3 dpl compared to extensive labeling of surrounding tissue in freshly lesioned animals (Fig. 1G-H). To further confirm that spread of dextran across the lesion site into the caudal spinal cord reflects anatomical continuity of the spinal cord and not dye diffusion across a still disconnected area, we analyzed dextran spreading in larvae that had been treated with DMSO control or IWR-1 to inhibit the Wnt/β-catenin pathway. We and others have recently shown, that Wnt/β-catenin signaling is required for anatomical and functional spinal cord regeneration in zebrafish larvae [1], [3]. The proportion of DMSO-treated control animals showing full length spinal cord labelling at 3 dpl matched that of untreated wild type levels (Fig. 2, and compare Fig. 1D), indicating that DMSO treatment did not affect reconnection of the spinal cord. In contrast, IWR-1 treatment for 3 days, starting immediately after lesion, significantly reduced the proportion of animals that showed dextran labeling in the spinal cord caudal to the lesion site by 84% compared to DMSO-treated controls (Fig. 2). This correlates with reduced axonal bridging of the lesion site, observed in *Xla.Tubb*:DsRED [4] live animals and by anti-acetylated immunohistochemistry after IWR-1 treatment as reported previously [1], [3]. Our dye injection data indicate that anatomical continuity is rapidly restored after complete spinal cord transection in zebrafish larvae in a Wnt/β-catenin signaling-dependent manner.

## 2 Experimental design, materials and methods

### 2.1 Animals

All fish were kept and bred in our laboratory fish facility according to standard procedures [5], and all experiments have been approved by the British Home Office (project license no.: 70/8805). We used the WIK wild type zebrafish strain.

### 2.2 Spinal cord lesion

Zebrafish larvae (3 days post-fertilization, dpf) were anesthetized in PBS containing 0.02% aminobenzoic acid ethyl methyl ester (MS222; Sigma-Aldrich) and transferred to a lid of a plastic Petri dish. Following aspiration of excess water, which placed the larvae in a lateral position, the tip of a sharp 30 G syringe needle was used to inflict a lesion in the dorsal trunk at the level of the anal pore. After surgery, larvae were returned to E3 medium for recovery and kept at 28.5 °C.

### 2.3 Dye injection and analysis

Larva were anesthetized in 0.02% MS222 and mounted in a lateral position in 1% low melting point agarose (Invitrogen, 16520-100) in E3 medium on a plastic Petri dish. Agarose-embedding is necessary to immobilize live larvae in a single plane. Embedded larvae were covered with E3 medium to prevent dehydration. 10 mg/ml Tetramethylrhodamine Dextran (MW: 3000; Molecular Probes) in PBS solution was directly applied to the rostral spinal cord at the level of the anterior end of the yolk extension by microinjection using a microinjection system, consisting of a stereomicroscope, a three-dimensional micromanipulator (World Precision Instruments, M3301R), and an automatic pressure microinjector (World Precision Instruments, SYS-PV820). For microinjection, custom-made glass injection needles were used. Microinjection needles were prepared from borosilicate glass capillaries (World Precision Instruments, 1B100F-4) using a micro-needle puller (Suttner Instruments Co., Model P-87) with the following parameters: heating cycle value: 410, pulling cycle value: 100, velocity value: 150, time: 200. Injection needles were filled with Dextran using a microloader (Eppendorf) and mounted on a PicoNozzle (World Precision Instruments, Kit 5430-ALL) connected to a micromanipulator. Before injection the tip of the injection needle was broken under a stereomicroscope using a pair of blunt forceps to produce a sharp end. Using the micromanipulator the injection needle was lowered to pierce the spinal cord of the larvae in a 45 °C angle at the level of the anterior end of the yolk extension. Dextran was released directly into the spinal cord, which resulted in immediate labeling of the same. Spread of dextran dye was monitored within a time window of 1 min using a fluorescence stereomicroscope (Leica). For imaging, larvae were released from agarose by use of fine forceps and mounted in 3% methyl cellulose solution in E3.

### 2.4 IWR-1 treatment

Drug treatments were performed according to the schematic timelines shown with the experiment. Up to 10 larvae were incubated in 7 ml of E3 embryo medium containing the Axin1 stabilizer IWR-1 (Sigma-Aldrich) to inhibit Wnt/β-catenin pathway activity. IWR-1 was dissolved in DMSO (6 mM stock) and used at a final concentration of 15 µM. This concentration efficiently suppressed lesion-induced upregulation of a transgenic reporter of Wnt/β-catenin-dependent transcription [1]. Larvae were kept in the dark at 28.5 °C and water was exchanged daily.

### 2.5 Sectioning

Larvae were fixed in 4% PFA-PBS at 4 °C overnight. After two brief washes in PBT (0.1% Tween-20 in PBS) PBS), larvae were embedded in 4% Agarose-PBS and sectioned at 50–10 μm using a vibratome. Sections were briefly rinsed in PBS stained for DAPI (Thermo Scientific) to visualize nuclei, followed by two washes in PBS and mounted in 75% Glycerol-PBS.

### 2.6 Statistics

All experiments were performed at least three times. To test for statistical significance we used Fischer's exact test. ***P<0.001. n.s. indicates not significant. Error bars always indicate the standard error of the mean (SEM).
