# Supplementary material for: Restoration of anatomical continuity after spinal cord transection depends on Wnt/β-catenin signaling in larval zebrafish
Source: Data Brief. 2017 Nov 4;16:65–70. doi: 10.1016/j.dib.2017.10.068 (PMC5709348; doi:10.1016/j.dib.2017.10.068)
Supplement: Supplementary file 1 — Supplementary material [file mmc1.docx]

**Declaration of interest**

Conflict of interest: none.
